# The development of general practice as an academic discipline in Germany - an analysis of research output between 2000 and 2010

**DOI:** 10.1186/1471-2296-13-58

**Published:** 2012-06-15

**Authors:** Antonius Schneider, Nadine Großmann, Klaus Linde

**Affiliations:** 1 Antonius Schneider Institute of General Practice, Klinikum rechts der Isar, Technische Universität München Orleansstrasse 47, 81667, Munich, Germany

**Keywords:** Research articles, Germany, Primary care, General practice, Academic performance

## Abstract

**Background:**

Governmental funding support is seen as a prerequisite for the growth of research in general practice. Several funding programs in the amount of € 13.2 Mio were introduced in Germany from 2002 to February 2012. We aim to provide an overview of publications reporting original data and systematic reviews from German academic family medicine published between 2000 and 2010.

**Methods:**

Publications were identified by searching the database Scopus and screening publication lists of family medicine divisions or institutes. Papers had to report original primary research studies or systematic reviews; at least one of the authors had to be affiliated to a German academic family medicine division or institute.

**Results:**

794 articles were included. The number of publications increased steadily starting from 107 in the period from 2000 to 2003, to 273 from 2004 to 2007, and finally to 414 from 2008 to 2010. Less than 25% were published in English in the first period. This proportion increased to 60.6% from 2008 to 2010. Articles published in a journal without impact factor decreased from 59.8% to 31.9%. Nevertheless, even in the most recent period only 31.6% of all articles were published in a journal with an impact factor above 2. The median impact factor increased from 0 in the first period to 1.2 in the last.

**Conclusions:**

The output of original research publications from academic research divisions and institutes for general practice in Germany greatly increased during the last decade. However, professionalism of German primary care research still needs to be developed.

## Background

Although being Europe’s most populated country Germany’s output of primary care research medicine by far lags behind that of the United Kingdom and the Netherlands [1]. This is at least partly due to the fact that health care politics and medical schools only lately and still hesitantly invest into an academic infrastructure for general practice and family medicine. For decades family medicine was not taught at German medical schools. In 1966, the first teaching assignment was awarded at the University of Freiburg to an external general practitioner (GP) and in 1976 the first chair for family medicine was founded in Hannover [2]. In 1978 family medicine became a mandatory course in the countrywide subject catalogue for medical schools but until today teaching is mostly done by external GPs without university position. Going along with the increasing evidence in the 90ies that primary care is the backbone of a rational health care system [3,4] the scientific expert advisory board (Wissenschaftsrat) demanded for academic primary care departments in German medical schools in 1999. This was also claimed by the expert advisory board for the development of the German health care system (Sachverständigenrat) in 2000 with respect to the health care needs of the German population. At this time there were only five chairs for general practice/family medicine established in the existing thirty-six medical faculties. As a consequence, the German Ministry of Education and Research funded the development of new and already existing academic departments with a total funding of € 13.2 Mio within the period of 2002 to 2012. In 2006, family medicine institutes or divisions were established at 13 medical faculties [2]. By January 2012 this number has risen to 25 institutes or divisions, including 19 chairs for general practice.

We performed a review of publication patterns carried out in a systematic way to provide an overview of the development of primary care research in Germany with respect to publications of original data and systematic reviews since 2000. This way the research needs for Germany in terms of content and methodology are to be identified.

## Methods

### Literature search

Publications were identified a) by searching the database Scopus (http://info.scopus.com/) and b) by screening publication lists of university groups. We selected the database Scopus as it comprises PubMed/Medline and also covers European journals in languages other than English which are rarely listed in PubMed/Medline. Using the affiliation field we searched for the respective university groups by combining the name of the city with the names of the division or institute (algorithm: AFFIL (Allgemeinmedizin city) OR AFFIL (General practice city) OR AFFIL (family medicine city) OR AFFIL (primary care city)). All references identified were imported into an Endnote database. In addition, we asked all German university groups to provide their publication lists.

### Study selection

To be included papers had to have been published between January 2000 and December 2010; had to report original primary research studies or systematic reviews (or protocols for such studies); and at least one of the authors had to be listed on the publication as affiliated to a German family medicine division or institute. One reviewer screened titles and (as far as available) abstracts of all Scopus search hits and excluded all clearly irrelevant publications (e.g. editorials, comments, correspondence etc.). The full text was obtained for all remaining articles. After the electronic search was run publication lists of the university institutes were screened for additional potentially relevant articles. These were obtained as full text, too, and references were entered manually into the Endnote database. The full texts were then checked for eligibility by one reviewer. A second reviewer was contacted in any case where selection was not straightforward.

### Data extraction

One reviewer extracted the following information (apart from reference information included in the Endnote file) from all included articles: language, total number of authors, number of authors with GP affiliation, GP institutions involved; whether first and last authors were from GP institutions, whether a statistician, a person from a non-GP department and person from another country was a co-author; design, subject, condition (if applicable) as free text and coded according to ICPC (International Classification of Primary Care). A second reviewer was contacted in any case of difficulties.

### Categorization

We categorized the included research publications similar to a scheme in a previous work [5]. On level 1 we separated primary research studies and systematic reviews, and for both of these categories whether articles already reported data or were protocols. Original research studies (including the respective protocols) were then subcategorized up to three further levels. On level 2 we differentiated quantitative studies, qualitative studies, mixed quantitative and qualitative studies, and instrumental research (e.g. questionnaire development). On level 3 quantitative and mixed methods studies were subdivided into intervention studies, diagnostic studies, observational studies, and others. Interventional studies were further classified on level 4 as either randomized or non-randomized, and observational studies as either cross-sectional, cohort or case–control studies.

**Figure 1:**
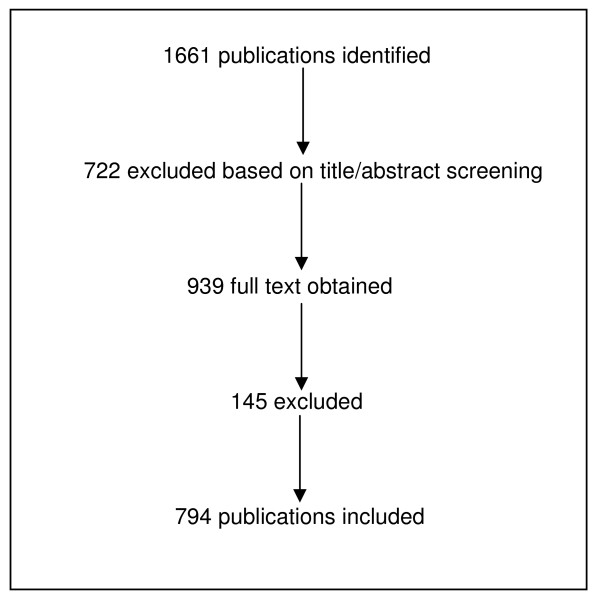
**Overview of included studies (classification according to **[2]**)**.

### Analysis

Discrete data (such as type of study design, study subject, type of affiliation or publication language) were summarized as absolute numbers and percentages, quantitative variables (such as impact factors, number of authors) as medians and ranges. To investigate changes over time three periods (2000 to 2003, 2004 to 2008 and 2008 to 2010) were defined. As our review covers a time span of 11 years it was not possible to have equally long analysis periods. We chose the three time periods post hoc as a compromise between similar length and statistical power to provide a sufficiently large number of publications of the time periods, thus allowing a better comparison of the publication activities with respect to the time course. The 2010 impact factor of journals listed in Thomson Reuters’ Journal Citation Reports was used as an estimate of the relevance of publications. For explorative comparisons of subgroups we used the Chi-squared-test and the Kruskal-Wallis-test depending on scale level.

## Results

The literature search identified a total of 1661 publications (after de-duplication). 722 publications were excluded based on the screening of abstracts and titles only, 939 were obtained in full text (see Figure 1). 794 articles met the inclusion criteria. 659 (83.0%) articles had been identified through the search in Scopus, 135 (17.0%) additional publications were identified by screening the publication lists of the university divisions and institutes. 46 of these 135 articles were actually not listed or listed inaccurately in Scopus, for 49 the affiliation field was incomplete, and for 40 articles there were other or unclear reasons.

**Figure 2:**
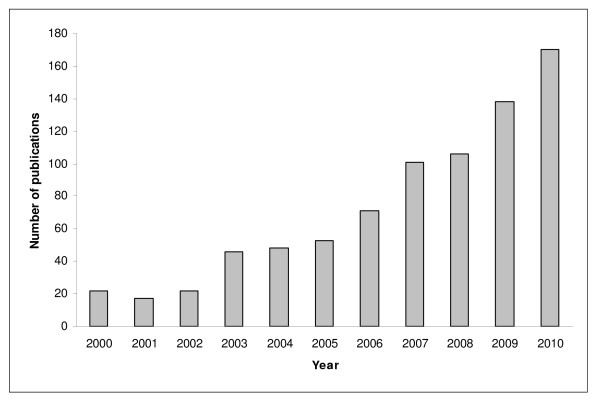
Flow chart.

401 (50.5%) articles were published in English, 391 (49.2) in German and 2 (0.3%) in French. The annual number of publications increased steadily over the years (Figure 2). Authors from two departments were listed on 36.9% of all publications (Heidelberg 173 and Göttingen 120 publications), six further departments contributed more than 50 articles each (a total of 390 publications (49%)), while the remaining 18 groups only contributed 234 (30.1%) publications (percentages add up to more than 100% as authors from more than one institution can be listed on a paper).

**Figure 3:**
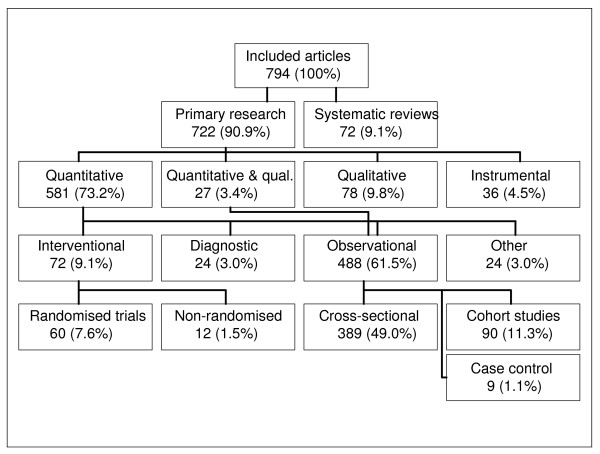
Number of research publications per year.

The categorization of articles is summarized in Figure 3. 722 (90.9%) publications reported original research (including 26 protocols) and 72 (9.1%) reported systematic reviews (2 protocols, respectively). Most original research was quantitative. The most frequent type of studies were cross-sectional studies (389, 49.0%), cohort studies (90, 11.3%), qualitative studies (78, 9.8%), and randomized controlled trials (60, 7.6%). The cross-tabulation of study subject and study design (Table 1) shows that a variety of approaches has been used in each area of research. Cross-sectional studies have most often been used in research on medical education (81.1%) and for exploring patients’ views (71.2%). Most of the clinical topics comprised psychological/psychosomatic complaints or diseases, cardiovascular, musculoskeletal and respiratory disorders or diseases (Table 2).

**Table 1:** Cross-tabulation of study subject and study type

	**Cross-sectional studies**	**Cohort/case control studies**	**Qualitative studies**	**Interventional studies**	**Systematic reviews**	**Instrumental research**	**Diagnostic studies**	**Other studies**
	**n = 389**	**n = 99**	**n = 78**	**n = 72**	**n = 72**	**n = 36**	**n = 24**	**n = 24**
Health services research (n = 314)	174 (55.5%)	35 (11.1%)	52 (16.6%)	39 (12.4%)	8 (2.5%)			6 (1.9%)
Clinical research (n = 216)	67 (31.0%)	43 (19.9%)	1 (0.5%)	26 (12.0%)	46 (21.3%)		24 (11.1%)	9 (4.2%)
Exploring patients’ views (n = 59)	42 (71.2%)	3 (5.1%)	13 (22.0%)		1 (1.9%)			
Methodology (n = 57)	16 (28.1%)	2 (3.5%)	1 (1.8%)	2 (3.5%)	2 (3.5%)	34 (59.6%)		
Medical education (n = 53)	43 (81.1%)	7 (13.2%)	1 (1.9%)			1 (1.9%)		1 (1.9%)
Various (n = 95)	47 (49.5%)	9 (9.5%)	10 (10.5%)	5551	15 (15.8%)	1 (1.9%)		8 (8.4%)

**Table 2:** Diagnostic groups of the published studies, according to ICPC-2 (n = 393 with a specific disease focus; few studies with more than one disease)

- Psychiatry	90 (22,9%)
- Circulatory	85 (21,6%)
- Muskuloskeletal	64 (16,3%)
- Respiratory	53 (13,5%)
- Metabolic, endocrinology	49 (12,5%)
- Digestive	20 (5,1%)
- Neurology	16 (4,1%)
- Urology	15 (3,8%)
- General	8 (2,0%)
- Skin	8 (2,0%)
- Male genital	4 (1,0%)
- Pregnancy	2 (0,5%)
- Female genital	2 (0,5%)
- Ear	1 (0,3%)

The comparison of the three pre-defined time periods (2000 to 2003, 2004 to 2007 and 2008 to 2010) shows relevant changes on several levels (Table 3). While the number of publications strongly increased from 107 in the first to 414 in the last (one year shorter) period, the number of university groups contributing to articles only increased from 19 to 25. There were no clear time trends regarding the use of specific study designs and preference for specific study subjects although chi-squared tests yielded statistically significant differences regarding cross-sectional studies and research on medical education (Table 3). Recent publications tended to have higher numbers of authors, more authors from GP divisions, and more often included a statistician or researcher from another country as co-author. While less than a quarter of publications were in English between 2000 and 2003, this proportion increased to 60.6% in the period of 2008 to 2010. Accordingly the proportion of articles published in a journal without an impact factor decreased from 59.8% to 31.9%. Nevertheless, even in the most recent period only 31.6% of articles were published in a journal with an impact factor above 2. The median impact factor increased from 0 in the first period to 1.2 in the last.

**Table 3:** Development of the publication output

**Years**	**2000-2003**	**2004-2007**	**2008-2010**	**p-value**	**All**
Number of publications	107	273	414		794
Number of groups with publication	19	25	25		28
Type of study					
- cross-sectional	52.3%	56.4%	43.2%	.003	49.0%
- cohort/case–control	13.1%	8.8%	14.7%	.07	12.5%
- qualitative	10.3%	8.1%	10.9%	.47	9.8%
- intervention	10.3%	10.3%	8.0%	.53	9.1%
- systematic reviews	9.3%	8.1%	9.7%	.77	9.1%
- instrumental/other	3.7%	6.2%	9.4%	.08	7.6%
- diagnostic studies	0.9%	2.2%	4.1%	.14	3.0%
Subject of study					
- health services research	43.0%	39.9%	38.4%	.68	39.5%
- clinical research	22.4%	26.0%	29.2%	.32	27.2%
- exploring patients’ views	10.3%	8.1%	6.3%	.33	7.4%
- methodology	5.6%	4.8%	9.2%	.07	7.2%
- medical education	2.8%	10.6%	5.1%	.004	6.7%
- various	15.9%	10.6%	11.8%	.36	12.0%
Authorship characteristics					
- total number of authors	4 (1-45)	5 (1-27)	5 (1-86)	< .001	5 (1-86)
- number of authors GP division	2 (1-7)	3 (1-11)	3 (1-9)	.001	3 (1-11)
- proportion of GP authors	0.75 (0.02-1)	0.67 (0.04-1)	0.67 (0.01-1)	.27	0.67 (0.01-1)
- first author from GP division	70.1%	74.0%	70.3%	.054	71.5%
- last author from GP division	60.7%	62.3%	67.6%	.22	64.9%
- statistician as co-author	7.5%	11.7%	19.1%	.002	15.0%
- co-author from other department	36.8%	43.6%	46.9%	.17	44.4%
- international co-author	11.2%	13.9%	23.4%	.001	18.5%
Published in English	24.3%	45.5%	60.6%	< .001	50.5%
Impact factor					
- median (range)	0 (0-10.0)	0.7 (0-16.7)	1.2 (0-36.4)	< .001	0.7 (0-36.4)
- without impact factor	59.8%	43.6%	31.9%	< .001	39.7%
- 0.001 to 2	35.5%	48.7%	58.0%		51.7%
- 2.001 to 4	9.3%	13.6%	21.5%		18.1%
- 4.001 to 10	3.7%	6.2%	8.2%		6.9%
- > 10	0.9%	1.5%	1.9%		1.6%

## Discussion

This analysis shows that the research output of publications with respect to original data and systematic reviews from academic research divisions and institutes of general practice in Germany has greatly increased during the last decade. Cross-sectional studies are by far the most frequently used study type accounting for almost half of all publications. While most research articles had been published in German language journals without impact factor in early years, more than half of the papers now are published in English language journals. However, publications in journals with high impact factors remain rare.

Governmental funding is demanded and seen as a prerequisite for the growth of primary care research [6]. Our results seem to support both such a demand and the success of the funding initiative of German politics mentioned in the introduction. This might be proved by our results, and it must be stated that the funding initiative of the government was successful. The increase of publication output is likely to reflect the professionalization of general practice on an academic level, which is in particular true with respect to international publications. The publication activity increased slightly at the beginning and has developed continuously since the introduction of the funding in 2002. Therefore, patience is necessary when the development of departments is monitored with respect to investment and efficiency. The funding mainly focused on four places (Frankfurt, Göttingen, Heidelberg, Marburg) with respect to the budget (more than € 1 Mio) with Heidelberg and Göttingen receiving the highest funding (> € 2 Mio), which was accompanied by the highest publication activity. Therefore, our results might reflect the direct association between funding and research productivity. At the same time it needs to be critically questioned if productivity and development will sustain as the targeted funding of primary care research is finished now in February 2012.

A welcome side-effect is the increased engagement of the academic general practice/family medicine in the development of guidelines and disease management programs. The German College of General Practitioners and Family Physicians (DEGAM) produced fifteen evidence and consensus based guidelines [7]. These are classified as S3 guidelines, which corresponds to the highest quality with respect to the graduation of the German Working Society of Scientific Medical Disciplines (Arbeitsgemeinschaft der Wissenschaftlichen Medizinischen Fachgesellschaften, AWMF) [8]. Beyond that, the DEGAM is involved in all seven national guidelines (Nationale Versorgungsleitlinie) [9]. The existing German disease management programs for chronic diseases (coronary artery disease, diabetes, asthma, chronic obstructive pulmonary disease) have been developed with participation of academic institutes. Therefore, the academic discipline of general practice/family medicine seems to be sustainably implemented in the structure of the German health care system.

Despite the increasing professionalisation and research output in recent years, Germany’s academic family medicine still has some way to go when compared to other countries with highly developed primary care research. The frequency of cross-sectional studies in our review is remarkable. It was pointed out in the Research Agenda of the European General Practitioner Research Network (EGPRN) that descriptive, cross-sectional surveys and attitude studies will not add much knowledge in most countries and settings; and there is a call for more interventional and randomised controlled studies (RCT), respectively [10]. Cross-sectional studies might indeed have been necessary to receive an overview of the specific primary care situation in Germany. E.g., we also have specialists working in primary care, and the contact rate in general practice is very high when compared with the UK or the Netherlands [11]. On the other hand, high quality studies like RCTs are expensive; and more funding might be necessary to realize more such trials. This might also partly explain the low number of high impact publications, in particular when compared with the research output of the research environment of other countries [1]. The total amount of public funding of primary care research in different countries is not easily available. Glanville et al. estimated the productivity of the primary care researchers by calculating the number of publications per billion dollar gross domestic research product spent on all research and development [1]. Thus the relation to the direct financial investment in primary care research remains unclear. It must be stated that there is no sustainable funding strategy for primary care research in Germany. The total funding of € 13.2 Mio (=16.8 Mio US dollars) over ten years appears to be low if compared with the gross expenditure on research and development (GERD) in Germany which was around 82 billion US dollars only for 2009 according to the OECD statistics [12]. As a consequence, German academic institutes of general practice are mostly small, in particular if compared with the Netherlands or UK. Another important barrier for international publication is the limited generalisability of the results of German health care studies to other countries.

When interpreting our findings several limitations have to be kept in mind. We did not search for and include articles by private GPs not affiliated with a medical school or research on primary care done by non-GP departments. Inclusion criteria were applied in a rather liberal manner. As a consequence, a number of articles (mainly process evaluations and developmental approaches in the area of health service research) presenting some original data have been included as primary research studies (mainly on the broad subject of health service research) for which it could be debated whether this is truly systematic research. Also, some articles included as systematic reviews used relatively vague methods. However, in all cases of ambiguity a second reviewer also assessed eligibility to ensure consistency of the selection process. A second reviewer was also involved if there were problems when categorizing a study. Due to the large number of articles and the limited resources available, the level of detail in the extraction had to be kept on a relatively low level. We could not include publications of 2011 in our review as it is very time-intensive to update our comprehensive search, obtain, select and extract an estimated number of over 200 publications. Besides that, we have not included literature which was published before 2000. However, the publication activity was low in the period before the funding by the Ministry of Education and Research, which started in 2002. As we covered a total of 11 years the three time periods compared in our analysis are not equal. While this is not optimal we believe it has little impact on the validity of our findings because the number of studies was higher in the last three years if compared with the previous four year intervals. A clear strength of our analysis is the reliance on full text articles. Borgers has repeatedly provided systematic bibliometric analyses of the publication output of Germany’s academic family medicine based on searches in the database Scopus (http://info.scopus.com/) [13-15]. He identified a total of 1130 publications in the period between 1998 and 2009, 683 of these being tagged as “original articles”. However, the bibliometric analyses rely completely on information included in Scopus and its formalized analysis options, and thus it was not checked whether an article contained original data. Therefore we had to exclude many of the articles which were identified by Borgers. Furthermore, potentially eligible publications were not only searched electronically in our study but also by direct contact with persons responsible for medical education and research on family medicine at all medical schools in Germany. Scopus searches alone missed 17% of eligible publications. For example, information on affiliation in Scopus is not regularly available for all authors, sometimes abbreviations are used (e.g. Dpt. Gen. Pract.), and in a few cases typing errors lead to missing a study. Overall, this suggests that analyses similar to ours should not exclusively rely on Scopus searches. We have used the journal impact factor as an estimate of the relevance of publications. While it is the measure most commonly used for this purpose it has several drawbacks [16]. A particular problem regarding publications from countries speaking languages other than English is that many journals which have national relevance are not covered by this instrument. Therefore, our analyses can only provide a crude estimate of the relevance of the included research articles. It has to be stated that solely counting impact factors only reflects a distinct part of the value of an academic discipline. There is a strong debate in this context [16,17], and the initiative to stratify primary care publications is important [18]. Reflective and narrative publications could not be included in a meaningful way. However, these might sometimes also be helpful for academic development.

## Conclusions

Research output with respect to publication of original data and systematic reviews from academic research divisions and institutes for general practice in Germany has greatly increased in the last decade. This development is likely to be due to a considerable extent to a targeted funding strategy of the Ministry of Education and Research. It remains a challenge to sustain in productivity and development as the funding period ended in February 2012. Professionalism needs to be developed further if German primary care research should compete successfully with other countries.

## Members of the DFG-network clinical trials in general practice

Jutta Bleidorn

Angela Buchholz

Annette Becker

Antonius Schneider

Attila Altiner

Frank Peters-Klimm

Guido Schmiemann

Eva Hummers-Pradier

Ildikó Gágyor

Jean-François Chenot

Jörg Haasenritter

Martin Scherer

Stefanie Joos

Wilhelm Niebling

Michael Kochen

## Competing interests

AS received funding from 2004 to 2006 for young academics for research in general practice (Nachwuchsförderung) from the Ministry of Education and Research.

## Authors’contributions

AS wrote the manuscript and helped with study design, NG searched and reviewed the articles and helped with writing, KL developed the study design, reviewed articles and helped with writing. "All authors read and approved the final manuscript.

## Pre-publication history

The pre-publication history for this paper can be accessed here:

http://www.biomedcentral.com/1471-2296/13/58/prepub
